# Tecovirimat: A journey from discovery to mechanistic insights in poxvirus inhibition

**DOI:** 10.1371/journal.ppat.1013140

**Published:** 2025-05-16

**Authors:** Xue Li, Zhengyang Pan, Leiliang Zhang

**Affiliations:** 1 Department of Clinical Laboratory Medicine, The First Affiliated Hospital of Shandong First Medical University & Shandong Provincial Qianfoshan Hospital, Jinan, Shandong, China; 2 Department of Pathogen Biology, School of Clinical and Basic Medical Sciences, Shandong First Medical University & Shandong Academy of Medical Sciences, Jinan, Shandong, China; Mount Sinai School of Medicine, UNITED STATES OF AMERICA

## Abstract

Tecovirimat (ST-246 or TPOXX) is an antiviral agent developed as part of a U.S. biodefense initiative aimed at addressing *Orthopoxvirus* infections, including smallpox and mpox. Although smallpox was declared eradicated in 1980, the potential for its reemergence as a biothreat persists due to illegal stockpiling and the possibility of laboratory synthesis. The F13 protein, which plays a critical role in the formation of extracellular viral particles, serves as the primary target for tecovirimat, inhibiting the transition from intracellular mature viruses (IMVs) to intracellular enveloped viruses (IEVs). Recent research indicates that tecovirimat stabilizes F13 homodimers as a molecular glue, effectively disrupting viral wrapping processes. However, the identification of tecovirimat-resistant mutations, particularly in immunocompromised individuals, highlights the urgent need for ongoing monitoring and the development of next-generation antiviral therapies. Investigating the structural dynamics of F13 and its interactions with tecovirimat may provide crucial insights into overcoming resistance mechanisms and improving therapeutic efficacy.

## Q: What is tecovirimat?

**A:** Tecovirimat (also known as ST-246 or TPOXX) is a small synthetic compound designed to combat poxvirus infections. It was discovered and developed as part of a biodefense initiative launched by the U.S. government in 2002 [1]. Smallpox, one of the most devastating infectious diseases in human history, claimed nearly 300 million lives during the 20th century alone. Although the World Health Organization (WHO) declared smallpox eradicated in 1980, the virus still poses a potential threat due to possible illegal stockpiling, natural reservoirs, or laboratory synthesis [2]. In addition, another *Orthopoxvirus*, the mpox virus, was declared a Public Health Emergency of International Concern (PHEIC) twice, in 2022 and again in 2024 [3]. As a result, the risk of outbreaks of both mpox and smallpox remains a significant concern for biosafety and public health.

**Fig 1:**
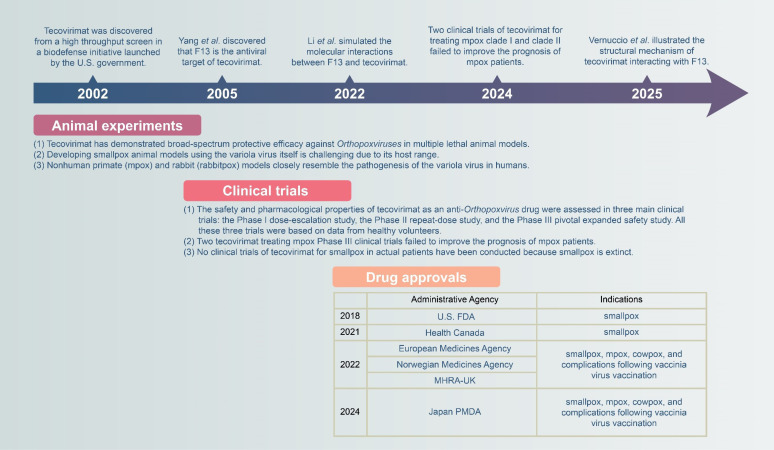
Key milestones in the development of tecovirimat and its mechanism against poxviruses.

In 2002, with no effective compounds available to combat smallpox, there was an increased interest in developing new small-molecule therapeutics [4]. In the biodefense initiative, a series of chemical compounds were evaluated for their ability to inhibit *Orthopoxviruses* through a high-throughput screening assay. Tecovirimat emerged as a leading candidate due to its balanced antiviral efficacy, metabolic stability, and low toxicity (Fig 1) [1].

Because smallpox has been eradicated, intentionally exposing volunteers to the variola virus is neither ethical nor feasible. The data from three main clinical trials—the Phase I dose-escalation study [5], the Phase II repeat-dose study [6], and the Phase III pivotal expanded safety study [7,8]—are entirely based on healthy volunteers. Thus, for tecovirimat treating smallpox, these are not typical clinical trials that involve actual patients and observation of therapy outcomes. The Phase I dose-escalation and Phase II repeat-dose studies evaluated the pharmacokinetics and safety properties of tecovirimat. Following these studies, the Phase III pivotal expanded safety study was a multicenter, randomized, double-blind, placebo-controlled trial that further assessed the safety, tolerability, and pharmacokinetics of tecovirimat [8]. In this context, the approval of tecovirimat is being expedited through an alternative development pathway based on human safety data and efficacy data derived from animal models, in accordance with the U.S. FDA Animal Rule [7].

The Animal Rule offers a pathway for the development of drug and biological products when human efficacy studies are ethically or feasibly limited. It allows a new drug product to be approved based on proven safety and robust animal study results under specific conditions [8]. Tecovirimat has demonstrated broad-spectrum protective efficacy against *Orthopoxviruses* in multiple lethal animal models, including those that can infect humans [8]. However, it should be noted that developing smallpox animal models using the variola virus itself is challenging since the host range of the virus is restricted to humans. Additionally, experiments involving the variola virus can only be conducted in high-protection biosafety level 4 (ABSL-4) laboratories. Therefore, it is crucial to create smallpox animal models using alternative pathogens that can closely mimic the critical characteristics of the variola virus. The pathogenesis of the mpox virus in nonhuman primates and the rabbitpox virus in rabbits closely resembles that of the variola virus in humans [8]. Consequently, nonhuman primate (mpox) and rabbit (rabbitpox) models are utilized to meet the animal rule requirements while developing tecovirimat as a potential therapeutic for smallpox [7].

Tecovirimat received its first approval from the U.S. FDA for the treatment of smallpox in 2018, followed by approval from Health Canada in 2021. Subsequently, in 2022, it was authorized by the European Medicines Agency (EMA), the Norwegian Medicines Agency, and the UK’s MHRA, and in 2024, by Japan’s PMDA for treating smallpox, mpox, cowpox, and complications related to vaccination against smallpox (Fig 1).

## Q: What is the drug target of tecovirimat on poxvirus?

### A:

The antiviral target of tecovirimat is the F13 protein. F13, which is homologous to the product of the cowpox virus *V061* gene, is a palmitoylated peripheral membrane protein encoded by the vaccinia virus *F13L* gene, and is essential for the formation of extracellular virus particles. Investigation of cowpox virus variants resistant to tecovirimat revealed a single amino acid mutation in the *V061* gene. Introducing this mutation back into the wild-type cowpox virus genome replicated the resistance to tecovirimat, indicating that the cowpox virus *V061* gene product is the target of the drug [4]. Furthermore, studies involving vaccinia virus with deletions in the *F13L* gene also exhibited resistance to tecovirimat [1].

## Q: What is the antiviral mechanism of tecovirimat?

**Fig 2:**
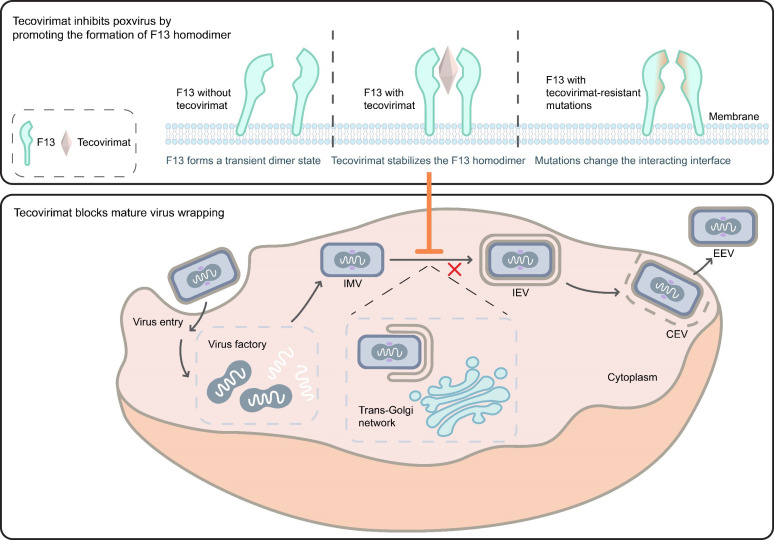
Mechanism of tecovirimat action against *Orthopoxvirus.* The upper panel illustrates the molecular mechanism by which tecovirimat interacts with F13. The lower panel depicts the stage affected by tecovirimat within the *Orthopoxvirus* life cycle. Tecovirimat binds to F13 and promotes the formation of F13 homodimers, thereby inhibiting viral wrapping. The area where tecovirimat exerts its effect is highlighted in orange.

**A:** *Orthopoxviruses* are double-stranded DNA viruses that replicate in the virus factory located in the cytoplasm following cellular entry. Four types of infectious virus particles are produced during this replication process: intracellular mature virus (IMV), intracellular enveloped virus (IEV), cell-associated enveloped virus (CEV), and extracellular enveloped virus (EEV) [9]. IMV is the first infectious particle assembled in the virus factory. After acquiring two additional membrane layers from the trans-Golgi network or endosomes, IMV transforms into IEV. The IEV then merges its outer membrane with the plasma membrane and remains attached to the cell surface, resulting in the formation of CEV. Finally, CEV released into the extracellular space becomes EEV, which, along with CEV, plays a crucial role in cell-to-cell spread and long-range dissemination of the virus [10,11]. F13, which is a phospholipase essential for viral wrapping, serves as the target for tecovirimat. Tecovirimat blocks the formation of IEV, thereby inhibiting the spread of progeny virus, rather than directly blocking viral replication (Fig 2) [1]. However, the specific mechanism by which tecovirimat interacts with F13 remains unclear.

In February 2025, Vernuccio and colleagues reported that tecovirimat acts as a molecular glue that promotes the formation of F13 homodimers [12]. Soluble F13 (sF13) was engineered by modifying two hydrophobic regions: removing the hydrophobic N-terminal tail (amino acids 2–5) and introducing five mutations in the hydrophobic membrane-interacting region (MIR). *In vitro*, sF13 was observed to form a homodimer stabilized by two helices and a β-hairpin. The interaction network includes hydrogen bonds with residues Y253, N259, N267, Y285, S292, and N300. The hydrophobic N termini and MIRs are positioned on one side near the center of the dimer, with the two phospholipase D active sites oriented outward. A large cavity of approximately 290 Å³ is formed between the two monomers within the homodimer.

In the presence of tecovirimat, the drug was found at the dimeric interface; however, due to a lack of characteristic electron density, the modeling of tecovirimat binding to the homodimer was completed using binding affinity calculations. The interaction network between tecovirimat and the homodimer includes polar contacts with Y258 and S292, as well as hydrophobic interactions with Y253, I262, I266, and Y285. A closer analysis indicates that tecovirimat binds to a transient dimer state, stabilizing it and thus promoting the formation of the homodimer (Fig 2) [12].

## Q: How do the tecovirimat-resistant mutations escape?

**A:** Tecovirimat has been widely used to treat mpox patients since the outbreak began in 2022. Research indicates that the reduction of tecovirimat activity is more pronounced in patients with compromised immune systems who are undergoing long-term therapy [3]. Tecovirimat appears to have a low resistance barrier, with multiple tecovirimat-resistant mpox virus strains reported. Specific mutations, including I327N, A295E, D294V, A290V, A288P, D283G, Y258C, N267D, and ΔN267, identified in clinical mpox virus strains, are located at the homodimer interaction interface. These mutations lead to a reduction in tecovirimat activity by preventing the dimerization of F13 that is normally induced by the drug. *In vitro*, in the absence of tecovirimat, the sF13^A295E^ mutant (sF13 with the A295E mutation) formed a homodimer resembling that of sF13^WT^, with Y285 forming a hydrogen bond with Q299 instead of N300, which would have been the case without the mutation. However, in the presence of tecovirimat, sF13^A295E^ regained its native conformation, with Y285 re-bonding to N300. Thus, tecovirimat-resistant mutations alter the interface of the F13 homodimer, impairing the dimerization induced by tecovirimat (Fig 2) [12].

Interestingly, only ten mutations, either isolated or in combination, were identified in the tecovirimat-resistant mpox virus strains. This is likely because most mutations near the dimer interface may cause a significant loss of viral fitness or even render the virus non-viable. Recombinant vaccinia viruses bearing the A295E mutation or the quadruple mutant (N267D, A288P, A290V, D294V) displayed lower viral titers compared to the wild-type (WT) recombinant vaccinia virus, suggesting a potential loss of viral fitness in the isolated tecovirimat-resistant mpox virus strains [12]. While the emergence of new tecovirimat-resistant mutants is unlikely, there is still a need for alternative therapeutic solutions for the existing mutants.

## Q: Is there anything else that needs to be discussed at the end?

**A:** In August and December 2024, two news releases from the U.S. National Institute of Allergy and Infectious Diseases (NIAID) reported setbacks in two clinical trials of tecovirimat for treating mpox clade I and clade II [13]. Tecovirimat failed to improve the prognosis of mpox patients in both trials. Consequently, tecovirimat may no longer be suitable for mpox treatment, although it may still be relevant for the treatment of smallpox. The urgent development of new drugs against mpox or *Orthopoxvirus* is essential. Since tecovirimat blocks viral spread rather than inhibiting replication, one possible reason for its failure in both mpox clade I and clade II trials could be that administering tecovirimat after the onset of mpox symptoms may be too late to prevent viral spread.

Although the structural analysis of the F13 homodimer and its complex with tecovirimat does not explain why tecovirimat failed in both trials for treating mpox, it may provide valuable insights for the development of next-generation drugs against poxviruses, enhance our understanding of *Orthopoxvirus* pathogenesis, and aid in preparedness for potential mutated strains. The cysteines at positions 185 and 186 in the F13 MIR undergo palmitoylation [14]; however, these cysteines were mutated to alanines in sF13 in the molecular mechanism study, resulting in a loss of palmitoylation capability. The current findings do not rule out the possibility that the removal of the hydrophobic N-terminal tail and the introduction of mutations in the MIR during the generation of sF13 could lead to structural deviations *in vivo*. Additionally, the mechanism by which tecovirimat prevents the IMV from wrapping, after promoting F13 homodimer formation, remains to be fully understood.
